# Spatial heterogeneity of dynamics of H1 linker histone

**DOI:** 10.1007/s00249-014-0962-0

**Published:** 2014-05-16

**Authors:** T. Bernas, W. Brutkowski, M. Zarębski, J. Dobrucki

**Affiliations:** 1 Nencki Institute of Experimental Biology, Polish Academy of Sciences, Warsaw, Poland; 2 Division of Cell Biophysics, Faculty of Biochemistry, Biophysics and Biotechnology, Jagiellonian University, Krakow, Poland

**Keywords:** H1 histone dynamics, Cell nuclei, Microscopy, FCS, RICS, FRAP

## Abstract

Linker histone H1 participates in maintaining higher order chromatin structures. It is a dynamic protein that binds to DNA and exchanges rapidly with a mobile pool. Therefore, the dynamics of H1 were probed in the nuclei of intact, live cells, using an array of microscopy techniques: fluorescence recovery after photobleaching (FRAP), raster image correlation spectroscopy (RICS), fluorescence correlation spectroscopy (FCS), pair correlation functions (pCF) and fluorescence anisotropy. Combination of these techniques yielded information on H1 dynamics at small (1–100 μs: FCS, RICS, anisotropy), moderate (1–100 ms: FCS, RICS, pCF) and large (1–100 s: pCF and FRAP) time scales. These results indicate that the global movement of H1 in nuclei (at distances >1 µm) occurs at the time scale of seconds and is determined by processes other than diffusion. Moreover, a fraction of H1, which remains immobile at the time scale of tenths of seconds, is detectable. However, local (at distances <0.7 µm) H1 dynamics comprises a process occurring at a short (~3 ms) time scale and multiple processes occurring at longer (10–2,500 ms) scales. The former (fast) process (corresponding probably to H1 diffusion) is more pronounced in the nuclear regions characterized by low H1 concentration, but the latter (slow, attributable to H1 binding) in the regions of high H1 concentration. Furthermore, some regions in nuclei (possibly containing dense chromatin) may constitute barriers that impair or block movement of H1 histones within short (<1 µm) distances.

## Introduction

Histone H1 interacts with linker DNA as it enters and exits nucleosomes (Thomas 1999). Owing to these interactions, linker histone H1 has been implicated in establishing and maintaining higher order chromatin structures (Brown et al. 2006; Carruthers et al. 1998; Fan et al. 2003; Zlatanova and Yaneva 1991b). Several in vitro studies demonstrated that histone H1 is involved in chromatin folding (Robinson and Rhodes 2006; Russo et al. 1995) and compaction (Nagaraja et al. 1995; Sato et al. 1999; Woodcock et al. 2006). It has been shown that drug-induced dissociation of H1 molecules from DNA induces large-scale chromatin aggregation (Wojcik and Dobrucki 2008; Wojcik et al. 2013). According to the dynamic model of chromatin structure, binding affinity of histone H1 to DNA may modulate gene activity (Bustin et al. 2005; Thomas 1999). Indeed, methylation of DNA correlates with changes in equilibrium between the unbound and DNA-bound H1 (Levine et al. 1993; McArthur and Thomas 1996). On the other hand, binding of H1 may influence acetylation of the core histones (Gunjan et al. 2001; Raghuram et al. 2009) and methylation of DNA (Rupp and Becker 2005). Biochemical evidence indicates that histone H1 may also regulate transcription (Juan et al. 1994; Zlatanova and Yaneva 1991a, b). Furthermore, specific H1 subtypes and their phosphorylated isoforms are associated with RNA splicing (Davie 1996). Histone H1 appears to exhibit no DNA sequence specificity and is unlikely to be involved in gene activation or repression directly (Bustin et al. 2005; Catez and Hock 2010; Catez et al. 2006). Depletion of multiple variants of H1 results in expression changes of only a few genes (Fan et al. 2005). On the other hand, global alterations of chromatin structure are detectable in this system (Fan et al. 2005).

It is important to note that, under in vivo conditions, H1 histone is but one element of the network that controls chromatin structure. This network also includes HMG protein family, heterochromatin protein 1 (HP1), P300/CBP associated factor (PCAF) and transcription factors (Catez et al. 2006, 2004; Phair et al. 2004). Changes in the concentration of other regulators may affect the binding equilibrium of H1 and therefore its interaction with chromatin (Catez et al. 2006, 2004). Moreover, some proteins, such as chaperone RanBP7, may bind H1 directly, modifying its affinity to DNA (Freedman et al. 2010). As a result, chromatin foci, characterized by a high concentration of histone H1, are formed. Summarizing, these data point to local variations in the involvement of H1 in the process of gene expression. H1 histones are very dynamic, as demonstrated using FRAP (Brown 2003; Lever et al. 2000; Misteli et al. 2000; Phair et al. 2004). It should be noted that the exchange is slow in case of core histones (Kimura and Cook 2001). Studies of large sections of cell nuclei (as opposed to localized measurements) indicate the existence of several H1 fractions of different mobility (Carrero et al. 2003; Phair and Misteli 2001; Phair et al. 2004). The presence of a stably bound H1 fraction has been described as well (Carrero et al. 2004). In addition, differences in mobility of H1 between chromatin compartments have been reported (Bancaud et al. 2009; Muller et al. 2009). These differences may be associated with molecular exclusion and diffusive hindrance phenomena (Bancaud et al. 2009). Hence, several models explaining the mobility of DNA-associated proteins have been proposed (Bancaud et al. 2009; van Royen et al. 2011). Nonetheless, the heterogeneity of H1 histone mobility and binding has not been analyzed systematically.

Understanding the complexity of histone-DNA interactions requires studying chromatin in vivo, in the nuclei of live cells, rather than ex vivo. The diversity of the mechanisms (e.g., constrained diffusion, transient binding) that determine histone H1 dynamics in vivo (Bancaud et al. 2009; Carrero et al. 2004; Phair et al. 2004) requires probing at small (1–100 μs: FCS, RICS, anisotropy), moderate (1–100 ms: FCS, RICS, pCF) and large (1–100 s: pCF and FRAP) time scales. Using this approach, we demonstrate that the dynamics of H1 comprises several spatial and temporal components. This heterogeneity is likely to represent diffusion of H1 and its immobilization at different binding sites within chromatin. It has been postulated that binding of proteins to DNA is enhanced under the conditions of high molecular crowding (Bancaud et al. 2009). Therefore, it is possible to envisage a scenario where binding of histone H1 to DNA is a component of a self-regulatory mechanism governing the compaction state of chromatin. In line with this notion, our data indicate that the dynamics of H1 are affected by its local concentration in the nuclei and thus, presumably, by the chromatin structure.

## Materials and methods

### Material preparation

HeLa cells stably expressing eGFP-tagged linker histone (H1.1) or free eGFP were kindly provided by Dr. T. Kanda (Kanda et al. 1998), Dr. H. Kimura and Prof. P.R. Cook (Kimura and Cook 2001). Both cell lines were cultured using the procedure established previously (Wojcik and Dobrucki 2008; Wojcik et al. 2013). Briefly, the cells were grown as monolayers in Dulbecco’s modified Eagle’s medium (DMEM) supplemented with 10 % fetal calf serum in glass-bottom petri dishes (MaTek). Where indicated, the cells were fixed with 1 % formaldehyde in PBS and equilibrated with DAPI (300 ng/ml in PBS).

### Microscopy imaging

Images of cell nuclei were registered using a Zeiss LSM 780 (and Leica SP5) confocal microscope with either 40× or 63× water immersion Plan Apo objective lens (NA = 1.2). The Zeiss system was equipped with two primary long-pass dichroic mirrors (405 and 488 nm) and a 32-channel GAsP PMT working in integration mode. The Leica system was equipped with an acousto-optical beam splitter (AOBS) and multialkali single-channel PMTs. Fluorescence of H1-eGFP was excited with 488 nm light (40 mW Ar ion laser at 1 % power, unless stated otherwise) and detected in the 490–560-nm range, whereas the fluorescence of DAPI was excited with 405 nm (20-mW diode laser) and detected in the 410–480-nm range. Unless otherwise indicated, the images (optical sections) were registered at 16-bit precision with a pixel size of 0.063 nm (256 × 256 pixels), pixel dwell time of 5.09 µs and the confocal pinhole set to 1 airy unit (at 530 nm). The imaging of live cells was performed at 37 °C in DMEM, with 5 % CO_2_. Fixed cells were imaged at room temperature.

### Fluorescence recovery after photobleaching (FRAP)

Time series of 138 optical sections (128 × 256 pixels) were registered in the middle of cell nuclei using the frame time of 240 ms. The FRAP protocol comprised 5 pre-bleach frames, 10 bleach scans (100 % of power of 488 line of Ar laser, total time 450 ms) and 133 post-bleach frames. The bleaching was performed in a vertical region (strip) encompassing the whole diameter of a nucleus. The strip widths varied from 0.66 to 2.66 µm. The series were corrected for background and normalized to the average intensity of the non-bleached nuclear area of the last five frames. The increase of average intensity was measured over the nuclear area covered by the bleaching strip to calculate the halftime of FRAP recovery. Moreover, the average intensity profiles over the nuclei were calculated for each post-bleach image in the series. The apparent H1 diffusion coefficients were then calculated using a strip photobleaching two-dimensional model, described in (Muller et al. 2009). Briefly, the following formulas were used for fitting of intensity profiles:

1 $$\begin{gathered} c\left( {y_{0} ,0} \right) = 1 - p\left( {\varTheta (y_{0} - a) - \varTheta (y_{0} + a)} \right) \hfill \\ c\left( {y,0} \right) = 1 - \frac{p}{2}\left( {{\text{erf}}\left( {\frac{a - y}{\sigma \left( t \right)}} \right) + {\text{erf}}\left( {\frac{a + y}{\sigma \left( t \right)}} \right)} \right) \hfill \\ \end{gathered}$$

where *c*(*y*_0_, 0) is the postbleach fluorescence intensity distribution, *c*(*y*, *t*) is the distribution in successive time steps (*t*), *Θ* is the unit step function with the halfwidth of the bleach depth *p*, erf is the error function and *σ*(*t*) its width.

The mean square displacement (*σ*(*t*)^2^) was plotted against time and the apparent diffusion coefficient extracted from the fit, as described in (Muller et al. 2009). FRAP kinetics were measured for 50 nuclei with each of the four strip widths.

### Raster image correlation spectroscopy (RICS)

Time series of 64 optical sections were registered with a pixel dwell time of 5.09 µs, line time of 3,050 µs and frame time of 810 ms. Images of fluorescence fluctuations were calculated by subtracting the moving average (9 frame window) from the raw series and rejecting the first and last four frames. A set of subframes with a 32 × 32 pixel window were generated from each frame by shifting the window in 8-pixel steps (in *x* and *y* dimensions). The spatial autocorrelation (ACF) functions (patterns) were then calculated for each subframe as described in (Digman et al. 2005, 2008; Digman and Gratton 2009) and averaged over the temporal dimension. The nuclei were segmented with global Otsu thresholding, and patterns (subframes) corresponding to <95 % overlap with a nuclear mask were rejected from further analysis. The remaining patters were then normalized (total intensity of 1) and clustered using the Knn algorithm (3 clusters) where the squared intensity difference between the central areas (16 × 5 pixels) of the patters was used as the distance metric. Median ACF patterns corresponding to the centers of the two most distant clusters were taken for two-step fitting of the H1 diffusion/binding model described in (Digman et al. 2008; Digman and Gratton 2009). First, the central ACF pattern region (corresponding to a line time shift of 0) was used to perform the initial one-dimensional fitting:

2 $$G_{D} \left( {\xi ,x} \right) = \frac{\gamma }{N}\left( {1 + \frac{{4Dt_{p} \xi }}{{\omega_{0}^{2} }}} \right)^{ - 1} \left( {1 + \frac{{4Dt_{p} \xi }}{{\omega_{z}^{2} }}} \right)^{{ - \frac{1}{2}}} \times \exp \left( { - \frac{{\left( {\frac{p}{{\omega_{0} }}} \right)^{2} x^{2} }}{{1 + \frac{{4Dt_{p} \xi }}{{\omega_{0}^{2} }}}}} \right)$$

where *G*_*D*_ is an (diffusion) autocorrelation function of the pixel time shift *ξ* and pixel coordinate *x*, *γ* is the shape parameter (0.35 for the Gaussian point spread function, PSF), *N* is the number of molecules, *t*_*p*_ is the pixel dwell time, *D* is the diffusion coefficient, ω_0_ and *ω*_*z*_ are the width and height of the the detection volume (PSF), as determined using FCS, and *p* is the pixel size.

The diffusion coefficient (*D*) within the range from 0.01 to 30 µm^2^/s was regarded as an indicator of the presence of the mobile fraction of H1. Therefore, constrained fitting of *D* was used in the second (final) analysis step, initialized with the value obtained in the first step. Otherwise, *D* = 0 was set as the initial value of the unconstrained algorithm. The final fitting was performed using a composite (diffusion and binding) two-dimensional model (Digman and Gratton 2009).

3 $$\begin{gathered} G_{D} = \frac{\gamma }{N}\left( {1 + \frac{{4D(t_{p} \xi + t_{l} \eta )}}{{\omega_{0}^{2} }}} \right)^{ - 1} \left( {1 + \frac{{4D(t_{p} \xi + t_{l} \eta )}}{{\omega_{z}^{2} }}} \right)^{{ - \frac{1}{2}}} \times \exp \left( { - \frac{{\left( {\frac{p}{{\omega_{0} }}} \right)^{2} (x^{2} + y^{2} )}}{{1 + \frac{{4D(t_{p} \xi + t_{l} \eta )}}{{\omega_{0}^{2} }}}}} \right) \hfill \\ G_{B} = A\exp - \left( {\left( {\frac{px}{{\omega_{0} }}} \right)^{2} + \left( {\frac{py}{{\omega_{0} }}} \right)^{2} } \right)\exp \left( {\frac{{4D(t_{p} \xi + t_{l} \eta )}}{{\tau_{B} }}} \right),\quad G_{DB} = G_{D} \times G_{B} \hfill \\ \end{gathered}$$

where *G*_*D*_ is an (diffusion) autocorrelation function of pixel time shift *ξ*, line time shift *η* and pixel coordinates *x* and *y*; *G*_*B*_ is a (binding) autocorrelation function with the characteristic time constant of the process *τ*_*B*_ and a relative amplitude *A*, and *G*_*DB*_ is the composite autocorrelation function (including diffusion and binding).

The RICS experiment was performed 5 times, while each of the experiments (clustered data sets) comprised 35 time series of images of cell nuclei. Fixed cells were used as a negative control for RICS pattern reconstruction.

The subframes with a 32 × 32-pixel window were then decomposed into complete series of 2D Kravtchouk polynomials (orders from 0 to 31, total of 1,024). Linear discriminant analysis (LDA) was then used to rank these polynomials and isolate those that corresponded to the best discrimination of subframes contributing different ACF patterns. Groups of the 256 polynomials that corresponded to the highest LDA discriminative power were isolated. Average values of these 256 polynomials were calculated in the two groups along with a single average for each of the remaining 768 polynomials.

### Steady-state fluorescence anisotropy

Series of ten optical sections through cell nuclei were registered with polarizers set alternately in S and P configuration. Steady-state H1-eGFP fluorescence anisotropy was calculated using the formula:

4 $$r = \frac{{I_{\text{P}} - GI_{\text{S}} }}{{I_{\text{P}} + 2GI_{\text{S}} }}$$

where I_P_ and I_S_ are the fluorescence intensities measured, respectively, in the direction parallel and perpendicular to the direction of polarization of the excitation light, respectively; *G* is the instrumental constant.

The instrumental constant (*G* = 1.849) was determined by imaging of 500 nM solution of rhodamine 6G in PBS (pH = 7.4) at 37 °C.

### Fluorescence correlation spectroscopy (FCS)

Calibration of the detection volume (PSF) was performed using 50 nM solution of rhodamine 6G (Gendron et al. 2008) in glass-bottom petri dishes (MaTek). The rhodamine was excited with 488 nm light (3 % of Ar laser) and detected in the 490–560 nm range, producing an average count rate of 32.5 kcps. The correction collar of the objective was adjusted to obtain the maximum fluorescence signal from H1-GFP in cell nuclei. The setting, which corresponded to 0.185-mm coverslip thickness, was kept constant for calibration and cell measurements. In these conditions, with *D* = 400 µm^2^/s, the waist diameter of the detection volume (*ω*_0_) was 0.26 µm, and its height (*ω*_*z*_) was 1.7 µm. Time-correlated single-photon counting (TCSPC) was used to register the fluorescence of H1-eGFP from single spots in cell nuclei in six consecutive 30-s intervals. The spots were placed in the areas characterized by low (20th–50th percentile of fluorescence intensity) or high (50th–80th percentile) local H1 concentration. In the first two intervals, significant photobleaching H1-eGFP was observed (typically 80 %). Therefore, only data collected in the last four intervals, where stable fluorescence of 200–500 cps was observed, were used in further analysis. The fluorescence ACF was calculated with 4-µs bins using Zen 2011 software (Zeiss, Poland) and described using the two-component diffusion model with triplet state relaxation:

5 $$G_{DT} \left( \xi \right) = G_{DT} \left( 0 \right)\left( {1 + \frac{{F\exp \left( { - \frac{\xi }{{\tau_{F} }}} \right)}}{1 - F}} \right)^{ - 1} \sum\limits_{i = 1,2} {A_{i} \left( {1 + \frac{\xi }{{\tau_{Di} }}} \right)^{ - 1} \left( {1 + \frac{\xi }{{\tau_{Di} \frac{{\omega_{z} }}{{\omega_{0} }}}}} \right)^{{ - \frac{1}{2}}} }$$

where *G*_*DT*_ is the autocorrelation function of time shift *ξ*, *τ*_*Di*_ is the relaxation times corresponding to H1 mobility, *A*_*i*_ is the the amplitudes of the components (1,2) corresponding to *τ*_*D*1_ and *τ*_*D*2_, and *F* is the fraction of the tripled component and *τ*_*F*_ the corresponding relaxation time.

Three FCS data were registered from 55 cell nuclei, 3 spots in a nucleus and 3–5 ACF curves from each spot. The median ACFs were calculated from 392 (low H1) and 271 (high H1) single measurements and used to fit the two-component model (Eq. 5). One may note that increasing the number of components to 3 and inclusion of the anomaly parameter did not produce significant improvement of the fit. On the other hand, numerical stability and robustness (e.g., sensitivity with respect to initialization) were decreased (owing to low signal-to-noise ratio, SNR).

### Spatial distribution of H1 and chromatin

Fluorescence of eGFP (histone H1) and DAPI (chromatin) was registered in optical sections through fixed cell nuclei. Gaussian pyramid reduction was applied to obtain a series of images at different resolution scales, from 512 × 512 pixels (0.083 µm per pixel) down to 16 × 16 pixels (2.656 µm per pixel). This spatial down-sampling (decrease in resolution) corresponded to an increase in the size of the smallest structures that contributed to the image.

Nuclei were segmented in the former images using Otsu thresholding, and the respective nuclear binary masks were scaled down (nearest neighbor algorithm) according to the frame size. The DAPI and eGFP fluorescence intensities were corrected for background and normalized to their average values on the nucleus-by-nucleus basis. Local concentration of H1 histone (eGFP fluorescence) in nuclei was plotted against that of chromatin (DAPI fluorescence) at several resolution levels, using the respective binary masks to reject background pixels. It should be noted that if the local concentration of H1 is proportional to that of chromatin, the contour plots (normalized to unity) should be symmetric around the diagonal. To detect possible deviations from this proportionality, the corresponding fractions of pixels located above and below the diagonal were quantified using an array of rectangular regions of interest (ROIs). The difference between the respective fractions was plotted against their ROI position along the contour plot diagonal. Integration of the positive and negative regions of these plots yielded the difference between the fractions of chromatin characterized by local concentrations of H1 (eGFP), which were higher or lower than predicted from the respective concentrations of chromatin (DAPI). Thus, populations of H1-rich and -poor (in relative terms) chromatin were quantified by integration of positive and negative values, respectively, in the plot of the difference versus position along the diagonal.

### Pair correlation function (pCF)

Time series of 100,000 line scans (64 pixels) were registered inside cell nuclei, with pixel size of 0.111 µm and dwell time of 5.09 µs. Subsets of 72,000 scans, where no H1-eGFP fluorescence photobleaching was detectable, were then taken for further processing. The moving average (window size of 8,191 scans) was calculated over time and subtracted from the raw data. ACF of the resultant fluorescence fluctuations was calculated, as described in (Hinde et al. 2012, 2011):

6 $$G\left( {\xi ,\delta x} \right) = \frac{{\left\langle {\delta I\left( {t,0} \right)\delta I\left( {t + \xi ,\delta x} \right)} \right\rangle }}{{\left\langle {I\left( {t,0} \right)} \right\rangle \left\langle {I\left( {t,\delta x} \right)} \right\rangle }}$$

where *G* is the pair correlation function (pCF) of time shift *ξ* and the distance between pixels along the scanned line *δx*; *δI* is the deviation of the instantaneous fluorescence intensity *I* from the average < > is the average over time *t*.

The pCF measurements were performed in 75 nuclei.

## Results

### Global mobility of H1 in cell nuclei

**Fig. 1:**
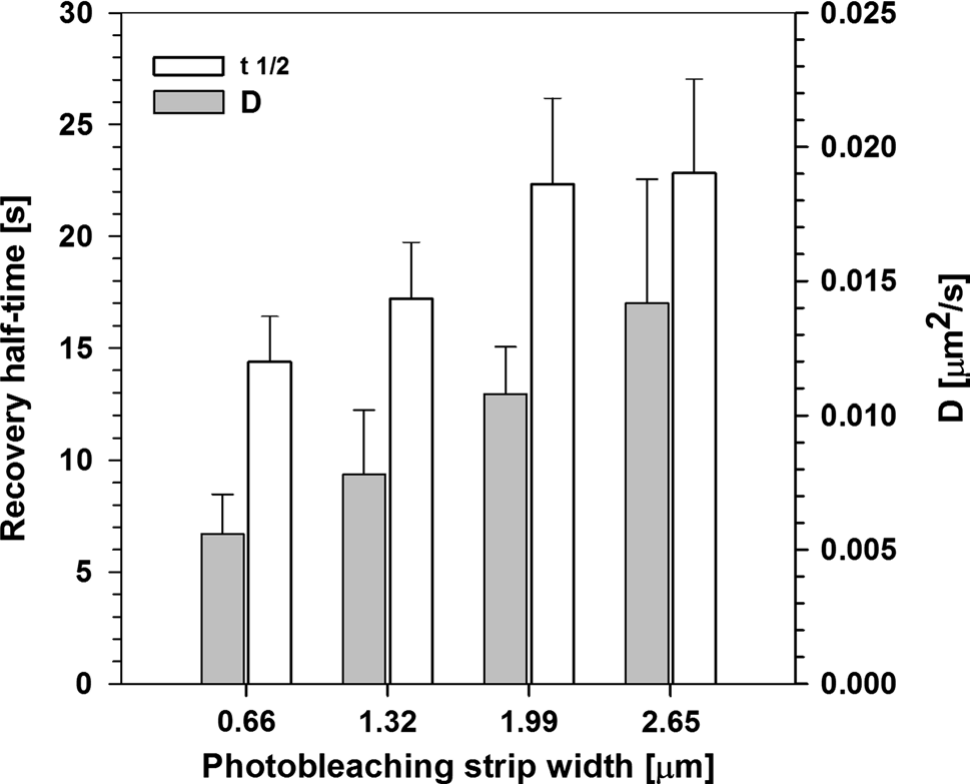
Dependence of fluorescence recovery halftimes (*t* ½, *left axis*, *light bars*) of H1 fluorescence and the diffusion coefficients (*D*, *right axis*, *light bars*) on the photobleaching region (*strip*) width in FRAP experiments. *Error bars* correspond to 95 % confidence intervals

Mobility of H1 linker histones was studied previously, in a global manner, using strip FRAP (Mueller et al. 2008, 2009), where bleaching regions of several widths, spanning across the whole nucleus, were used (see “Materials and methods”). In our experiments the halftimes of fluorescence recovery increased with the width of the bleaching region (Fig. 1), indicating that diffusion of H1 contributed to the recovery of its fluorescence. However, this increase was smaller than expected if movement of H1 were to be limited by diffusion only. Thus, one may postulate that an exchange between the mobile and the immobile H1 fraction occurred at the time scale of the FRAP experiment, limiting the observed recovery speed. The detailed analysis yields the apparent diffusion coefficient ranging from 0.011 ± 0.03 µm^2^/s (strip width of 0.66 µm) to 0.019 ± 0.05 µm^2^/s (strip width of 2.65 µm). These values correspond, respectively, to the residence times within focal volume from 6,145 ms down to 3,558 ms, respectively, and are two orders of magnitude longer than predicted for diffusion of molecules of a similar size in cell nuclei (Carrero et al. 2004). It should be noted that a significant fraction of H1 (~25 %) is immobile at the time scale of the FRAP experiment (up to 50 s). No immobile fraction was detectable when free eGFP was in the cell nuclei was studied. Conversely, more than 95 % recovery of fluorescence was observed within the first post-bleaching frame in these control experiments. Therefore, one may postulate that nuclear H1 dynamics is determined by long-time-scale entrapment of histone molecules in chromatin. Binding of H1 to DNA is a likely explanation of this effect. On the other hand, the increase in the apparent diffusion coefficient when FRAP measurements are performed at larger scales (Fig. 1) indicates that faster processes (e.g., diffusion) may contribute to the H1 dynamics. One should also note that FRAP experiments yield results averaged over regions of chromatin of different structure (degree of compaction). Thus, to explore the putative heterogeneity of H1 dynamics, the mobility of H1 histones was further probed locally in cell nuclei at several temporal and spatial scales.

### A pattern of subnuclear H1 histone mobility

**Fig. 2:**
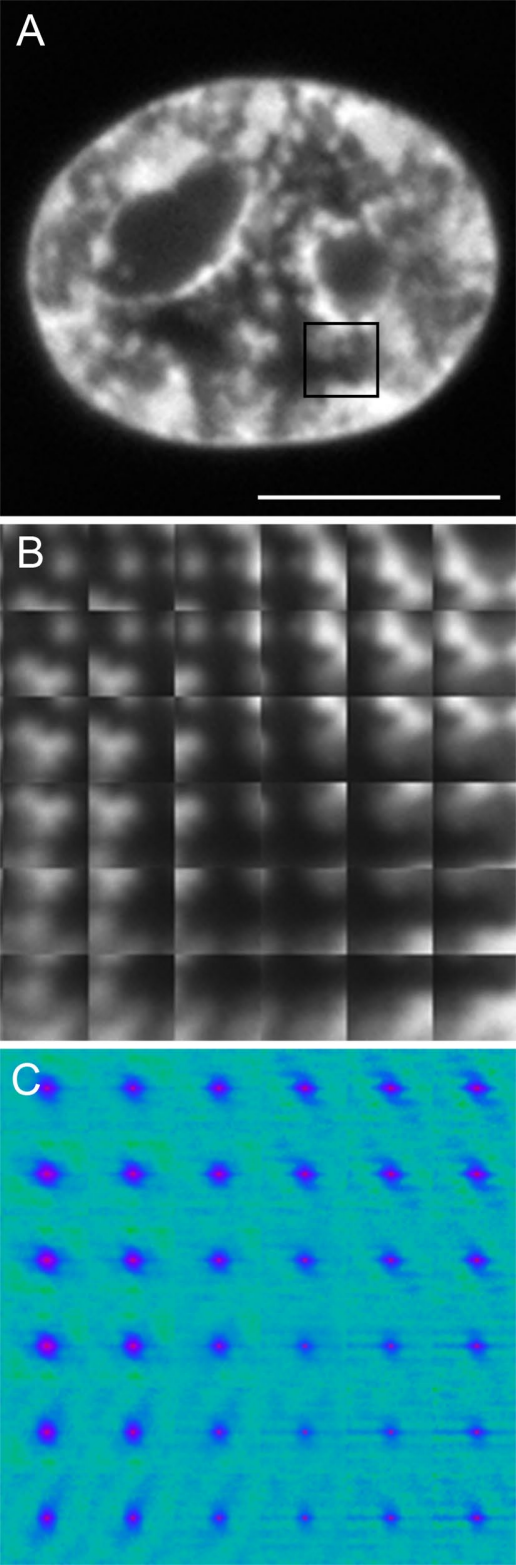
Example of nuclear distribution of H1 fluorescence, with a region marked with the rectangle (**a**). A magnified set of 32 × 32-pixel intensity subimages (**b**), corresponding to the region in *panel* **a**. The patterns of autocorrelation (RICS) functions of H1 fluorescence fluctuations (**c**), calculated in the set of windows shown in *panel* **b**. *Scale bar* 10 µm

**Fig. 3:**
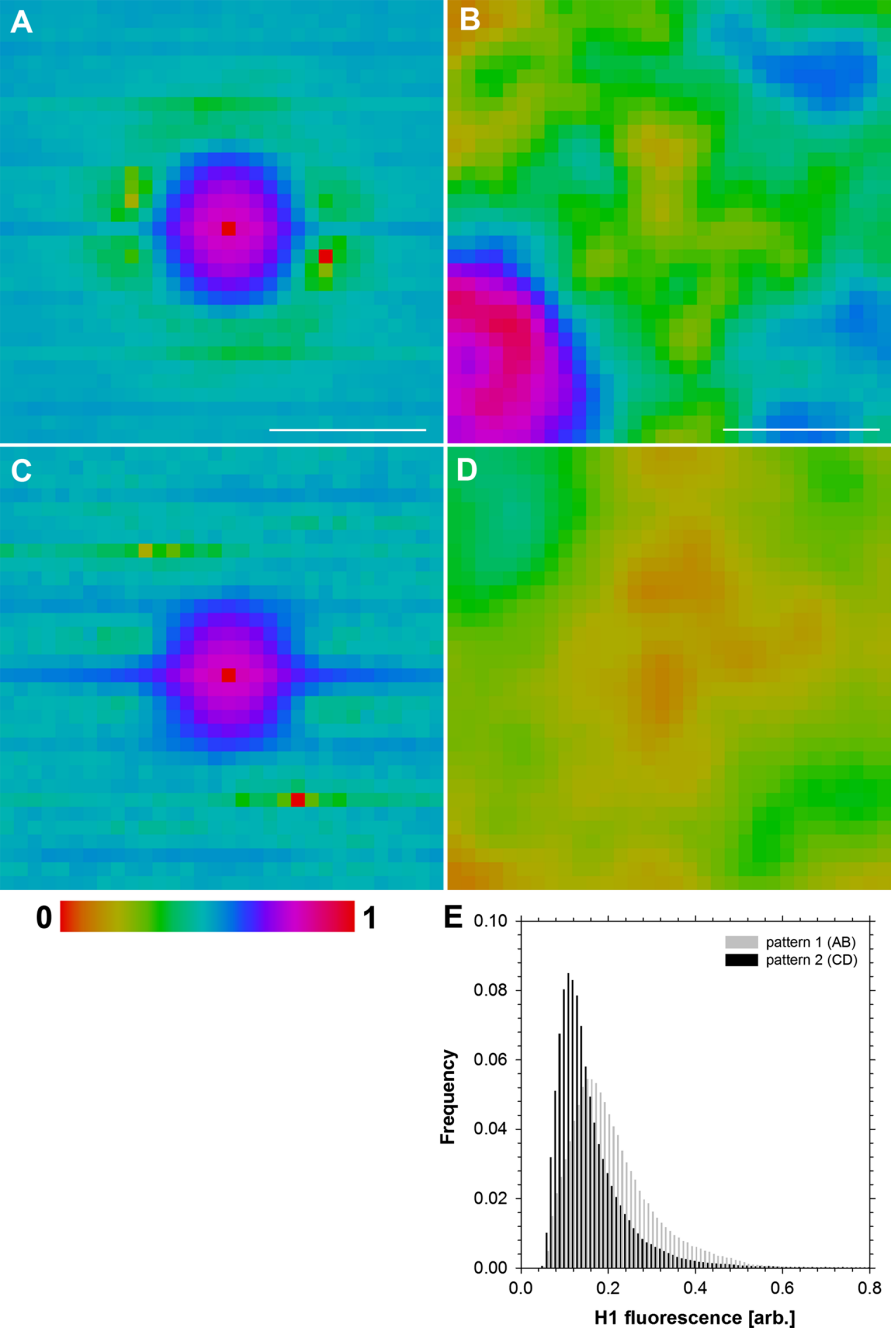
Patterns of autocorrelation (**a**, **c**) of H1 fluorescence fluctuations (RICS), revealing the transiently bound H1 fraction (**a**, **c**, central peak) and mobile H1 fraction (**c**, *horizontal line*). The corresponding spatial distributions of H1 (BD) were reconstructed with texture clustering. Fluorescence intensity *histograms* corresponding to both pattern types (**a**, **c**) are shown in *panel* **e**. *Scale bar* 10 µm

Mobility of histone H1 was studied using RICS (see “Materials and methods”), which generated an autocorrelation function of H1 fluorescence fluctuations (ACFs). The function was calculated in a set of partially overlapping windows covering the whole area of the nucleus (Fig. 2a). An example set of these windows (embracing a part of the nucleus—a frame in Fig. 2a), which was used to calculate ACF (Fig. 2c), is shown in Fig. 2b. An inspection of the complete data set (covering several nuclei) indicates that different ACF patterns may be present in a single nucleus (Fig. 2c). To verify this hypothesis, the ACFs corresponding to several nuclei were clustered using the Knn algorithm (“Materials and Methods”). The analysis reveals two main RICS ACF patterns (Fig. 3a, c). Both patterns contain a major component corresponding to a central maximum (characterized by an ellipsoidal shape). However, the patterns differ with respect to the presence (Fig. 3c) of a minor component corresponding to a horizontal line. This shape of the pattern indicates that two fractions of H1, characterized by a long and a short residence time, respectively, may be present. Therefore, a diffusion and binding model (Digman et al. 2008; Digman and Gratton 2009) was used to analyze the patterns, yielding the long residence (binding component) time of 22 ± 5 ms (Fig. 3a, c) and 18 ± 6 ms (Fig. 3c) and the short residence (diffusion component) time of 3.2 ± 2.1 ms. The latter may be converted to the diffusion (*D*) coefficient of 21 ± 15 µm^2^/s, using the volume established with FCS calibration (see “Materials and methods”).

Inspection of RICS ACF patterns (Fig. 2c) also suggests that their nuclear distribution may be non-random. To verify that notion, the respective nuclear subimages (frames) of H1 fluorescence intensity (Fig. 2b) were grouped according to the ACF cluster membership. The subimages were decomposed into series of 2D Kravtchouk polynomials (Bayraktar et al. 2007) to isolate these elements of H1 nuclear distribution, which corresponded to the two different mobility patterns of this histone. To that end, the polynomials that contributed the most to the difference between these two groups were chosen to generate semi-synthetic H1 intensity distributions (Fig. 3b, d). Comparison between these images (representative nuclear textures) indicates that the fraction of H1 characterized by the short residence time (Fig. 3c) is present in the nuclear regions where the concentration of the H1 histone is low (Fig. 3d). Conversely, the long residence time of H1 was observed in nuclear regions characterized by a high concentration of H1 (Fig. 3b). This notion is confirmed by a comparison between raw H1 fluorescence intensity distributions (Fig. 3e) corresponding to the two types of ACF (Fig. 3a, c). Not surprisingly, both spatial distributions of H1 intensity are not uniform (Fig. 3b, d) as the window, (32 pixel, 2.00 µm) used to calculate the respective ACF patterns is larger than the resolution limit of the microscope. Consequently, the window may contain regions corresponding to different H1 concentrations, resolvable with confocal microscopy.

### Mobility of H1 vs. concentration of the histone

**Fig. 4:**
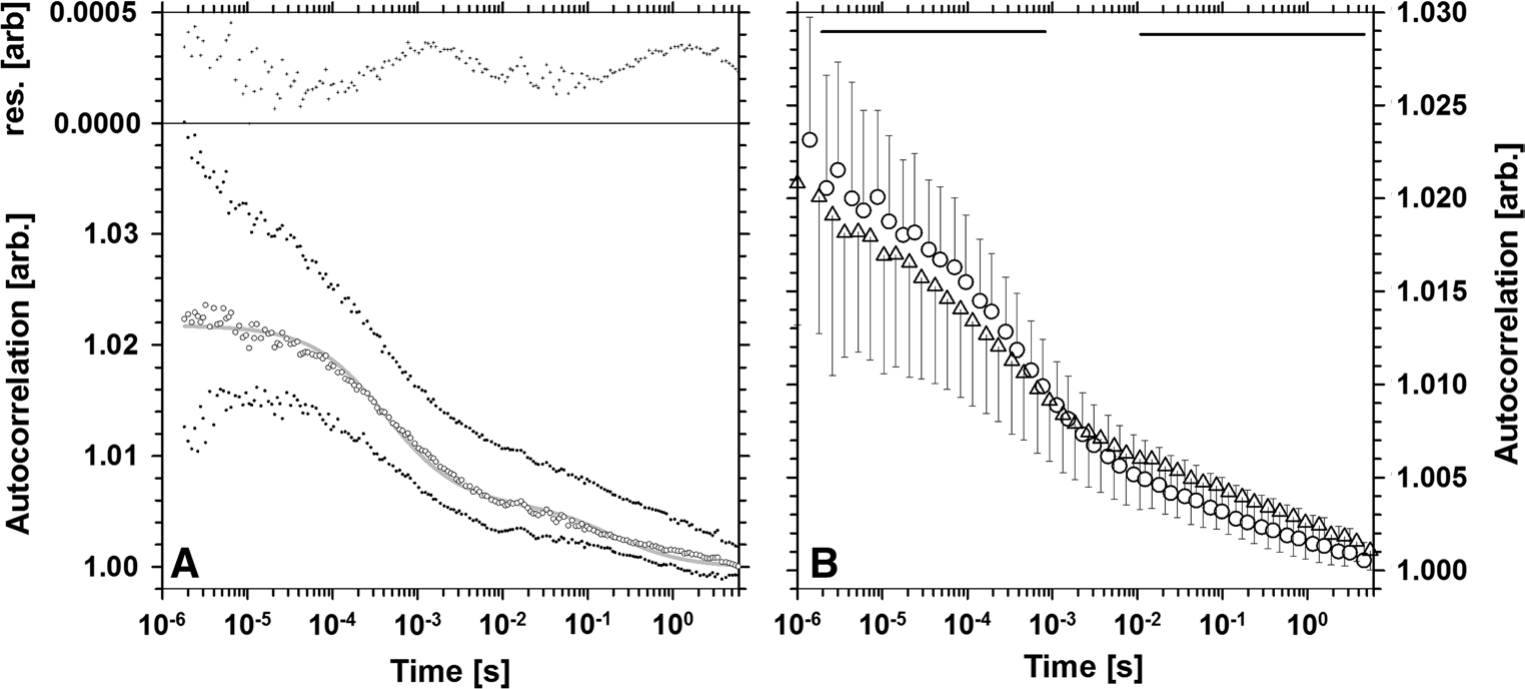
Fluorescence autocorrelation curve (**a**) corresponding to regions of low concentration of H1 shown as median (*circles*) with 5th and 95th percentiles (*dots*). The fitted two-component model is represented with a *solid gray line* (*bottom part*) and the residuals with crosses (*top part*). Comparison of mean autocorrelation curves (**b**) corresponding to areas of low (*circles*) and high (*triangles*) H1 concentration. *Error bars* correspond to the standard deviation. The regions where the two curves are different (*p* < 0.01) are marked with *solid horizontal lines*

The pattern of H1 mobility was probed using FCS in nuclear areas characterized by a low and high concentration of this histone. In accordance with RICS results, the FCS autocorrelation function (ACF) exhibits both rapidly (*τ* = 0.78 ± 0.06 ms, *D* = 21.1 ± 1.9 µm^2^/s) and slowly (*τ* = 298 ± 50 ms) decaying components (Fig. 4a). The residuals of the fitted curve (two-component model; see “Materials and methods”) were distributed in non-random fashion (Fig. 4a, insert). Thus, the time corresponding to the fast component might have been underestimated, while its slow counterpart overestimated. Nonetheless, these two components were clearly present in the areas of both high and low H1 concentration (Fig. 4b) in the cell nuclei. The fast component was more pronounced in the nuclear regions of low H1 concentration than in the regions characterized by high H1 concentration (Fig. 4b). The opposite situation was observed in the case of the slow component. One should note that, in contrast to RICS measurements, the immobile fraction of H1 was eliminated in FCS experiments (see “Materials and methods”).

**Fig. 5:**
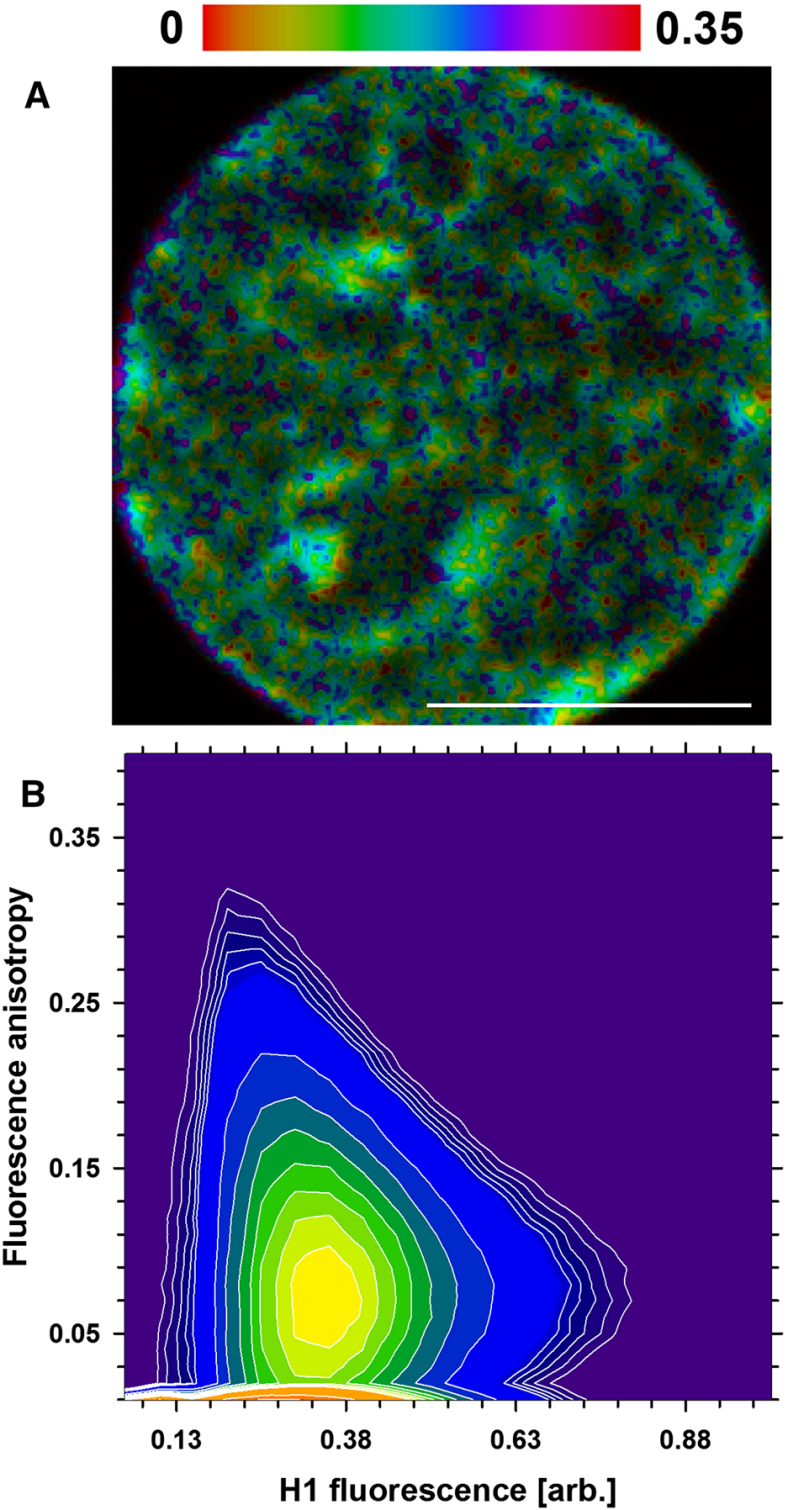
Rotational mobility of H1 in cell nuclei. Distribution of anisotropy is shown in color scale (*top*), whereas the H1 concentration is rendered with intensity (**a**). Relationship of H1 concentration (fluorescence intensity) and anisotropy is shown as contour plot (**b**) normalized to unity. *Scale bar* 10 µm

A possible heterogeneity of H1 mobility in cell nuclei (probed previously with RICS and FCS) was also studied with fluorescence anisotropy (Banerjee et al. 2006). The rotational mobility of H1 was found to vary in different regions of the nucleus (Fig. 5a); that is, low mobility (high anisotropy) was observed in the regions of high local H1 concentration. However, some of these regions also exhibited low anisotropy (Fig. 5a). A plot of anisotropy versus H1 concentration (Fig. 5b) indicates that in the majority of nuclear regions H1 histone exhibited high rotational mobility (low anisotropy). The lowest H1 mobility (high anisotropy) was detectable in areas corresponding to moderate H1 concentrations (Fig. 5b). These regions tend to be located between areas of low and high H1 concentration. In should be noted that the Pearson correlation between these two parameters, while low (*r* ^2^ = 0.1 ± 0.05), is still significantly different from 0.01 ± 0.03 (*p* < 0.03), which was measured for a free GFP under these conditions. This observation suggests that H1 rotational mobility was restricted to a large degree in the whole nucleus.

### Correlation of local abundances of H1 and chromatin

**Fig. 6:**
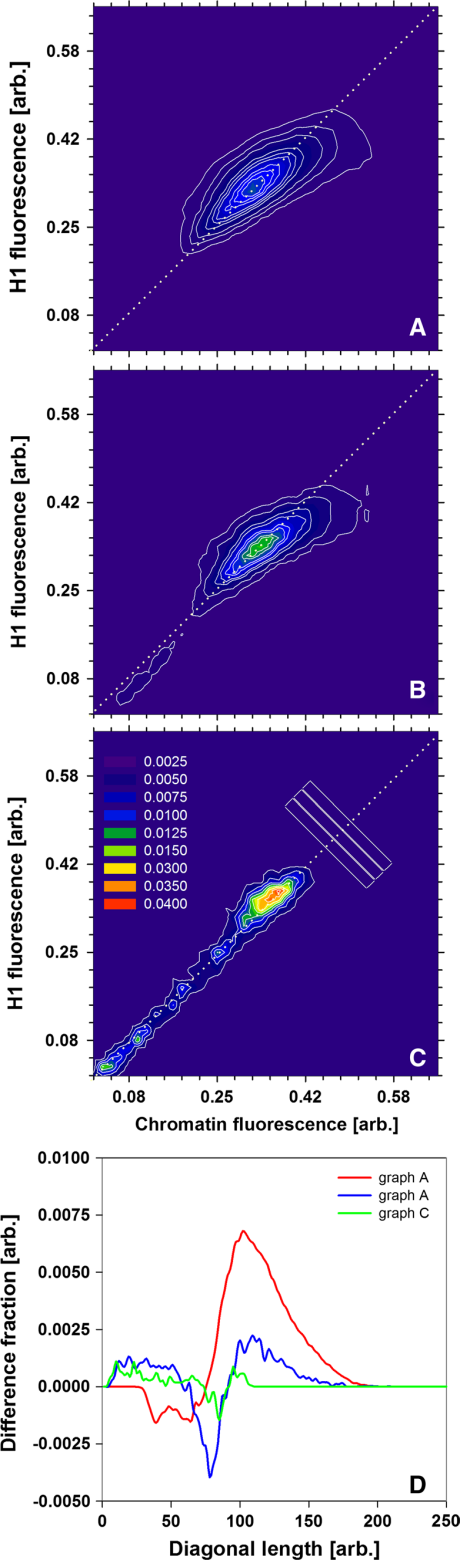
Correlation of local concentrations of H1 and chromatin (DAPI) measured at 0.166 µm (**a**), 0.664 µm (**b**) and 2.656 µm (**c**) spatial resolution, generated with Gaussian pyramid. The pixel density (normalized to 1) is represented with contour plots. Difference (*D*) between the (fractional) pixel density above and below the diagonal (*gray dotted line*) was calculated in rectangular regions (marked in *panel* **c**) for the data shown in *panel* **a** (*red*), **b** (*green*) and **c** (*blue*)

Heterogeneity of H1 mobility in cell nuclei suggests that no direct proportionality may exist between the local histone concentration and the state of compaction of chromatin. These two local parameters were compared at several levels of spatial resolution (see “Materials and methods”). The local concentrations of H1 histone and chromatin (DNA, labeled with DAPI) are strongly correlated, as shown in Fig. 6. The respective coefficient increased with decreasing resolution level (i.e., an increasing size of the smallest structures that contribute to the image), ranging from 0.8 ± 0.05 (0.166 µm pixel size, Fig. 6a) to 0.9 ± 0.06 (2.656 µm pixel size, Fig. 6c). It is important to note that the plots corresponding to high and intermediate spatial resolutions (Fig. 6a, b) are non-symmetric around the diagonal (Fig. 6d). This notion indicates the presence of regions of concentrated chromatin in which the abundance of H1 is relatively low (Fig. 6a, b, d). These regions occupy only a minor fraction of the nuclear volume (~3.4 %), as determined by integration of the respective fractional difference curves (Fig. 6d). On the other hand, regions with a low concentration of chromatin but relatively high concentration of H1 were also detected (Fig. 6a, b, d). They occupied an even smaller fraction of the nuclear volume (~0.4 %). These fractions were not detectable at low resolution (Fig. 6c, d). Thus, one may estimate that the diameter of the regions characterized by a low concentration of H1 and a high chromatin content falls between 1,400 and 2,800 nm. We postulate that they represent dense heterochromatic regions in which the concentration of H1 is relatively low.

### Barriers of H1 mobility

**Fig. 7:**
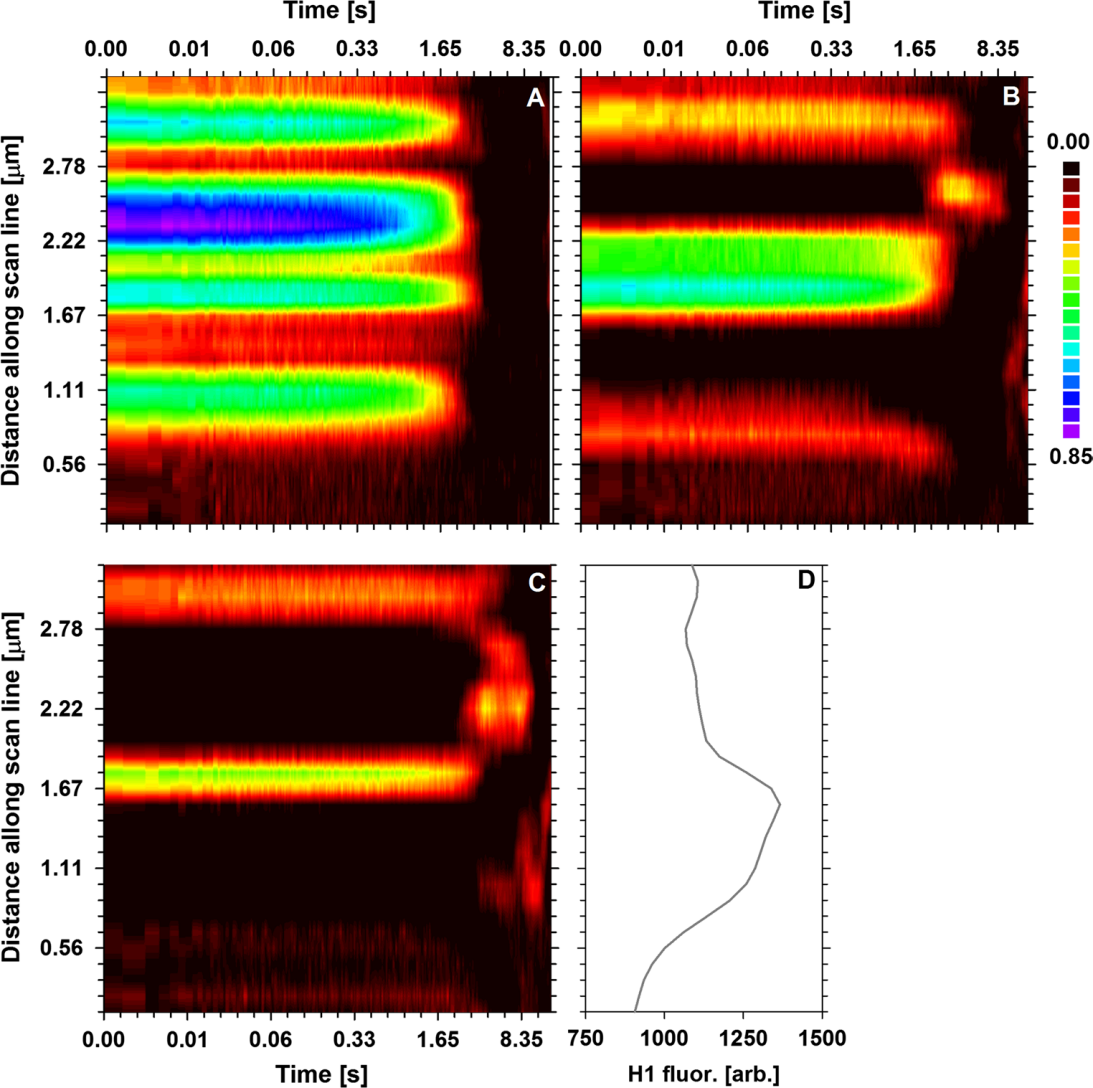
Pair correlation functions (pCF) corresponding to scanning along an 8.25-µm line, calculated at the offset of 0 nm (**a**), 446 nm (**b**) and 890 nm (**c**). The amplitude of pCF is rendered with the heat map scale (from 0 to 1). The intensity profile along the scanned line is shown in *panel* **d**

The ability of H1 to move between different regions of nuclei was investigated with pair correlation functions (pCF). The shape of pCF at the offset of 0 nm (corresponding to a simple ACF) demonstrated that the residence time of the mobile H1 fraction fell between 536 and 1,100 ms (5th and 95th percentiles of the distribution), depending on the region (Fig 7a). This value may correspond to the slowly decaying component, detectable with FCS. On the other hand, the H1 histone seems to be virtually immobile in certain areas. The analysis of cross-correlation between different points along the scanned line (Fig. 7b, c) indicates that certain areas where little mobile H1 is detected (at the offset of 0 nm) may nonetheless be crossed by the histone molecule. This process is longer (2,500–4,000 ms) than the time corresponding to the residence of the mobile H1. Conspicuously, this value is similar to the average residence time measured with FRAP. Moreover, some regions of immobile H1 constitute absolute barriers to its mobility (Fig. 7b, c). It is also worth noting that there was no obvious correlation between the presence of a mobile H1 histone and its local concentration (Fig. 7d).

## Discussion

Global (embracing the whole or large areas of the nucleus) measurements of H1 dynamics with FRAP indicates that approximately 25 % percent of the histone H1 pool is strongly bound (at the time scale up to 50 s) while the rest is moving with the effective diffusion coefficient D (~0.015 µm^2^/s). This value is significantly smaller than the *D* = 40 µm^2^/s predicted for free diffusion of H1-GFP in the nuclear environment (Carrero et al. 2004). This result can be attributed to a rapid binding/dissociation events that occur on the time scale of a FRAP experiment, thereby slowing down the histone movement. Molecular crowding arising from compaction of higher order chromatin structures may provide an alternative explanation of this diffusive hindrance (Bancaud et al. 2009). However, only a minor fraction of free eGFP (approximately 3 % of the total), characterized by having a recovery slower than that of diffusion-controlled FRAP, was detected. The presence of this fraction might have been a result of a transient immobilization of eGFP in chromatin caused by molecular crowding. However, the characteristic time of this putative effect (*τ* = 1.0 s ± 0.2) was too short to account for the kinetics of the slower FRAP recovery (*τ* > 6.5 s) of H1 histone. Moreover, the increase in the FRAP recovery halftime (*t* ½) with the size of the bleaching region is consistent with the former hypothesis (binding), indicating that the H1 mobility could be described using an effective diffusion model (Lever et al. 2000; Sprague et al. 2006, 2004). It may seem surprising that the effective diffusion coefficient increased with *t* ½ in the FRAP experiments. However, one may postulate that the equilibrium concentration of free H1 was achieved during photobleaching of a narrow strip (thereby reducing the effective bleach depth), but for wider strips, the process continued into a recovery phase of the FRAP experiment (contributing to an increase of the strip profile width). This notion, corroborated by the analysis of the fitting parameters, indicates that the H1 diffusion over larger distances in the nucleus may be impeded (as discussed below). The magnitude of the diffusion coefficient measured in this study was lower than those reported by other workers (Carrero et al. 2004; Stasevich et al. 2010). This might be explained in part by the fact that different cell models were used—3T3 fibroblasts (Stasevich et al. 2010) or SK-N-SH neuroblastoma (Carrero et al. 2004, 2003) in the previous studies, whereas HeLa (epithelial origin) cells were used here. It is possible that H1 histone mobility is dependent on the cell type. It should also be noted that the *t* ½ of H1 FRAP recovery measured previously in our group using HeLa cells (Wojcik and Dobrucki 2008; Wojcik et al. 2013) was longer than those reported in other cell models (Brown et al. 2006; Lever et al. 2000; Phair et al. 2004). One should also note that the FRAP results represent an average over chromatin regions characterized by a varying concentration of H1 and presumably by heterogeneous spatial structure. This hypothesis was further verified using RICS to probe the local heterogeneity of H1 mobility. The results indicate that the mobility is affected by entrapment of the histone, which occurs everywhere in the cell nucleus. Binding of H1 to DNA is a plausible explanation of this phenomenon. The time of residence (binding) of H1, determined using RICS, is markedly lower than that measured with FRAP by other workers (Carrero et al. 2004; Phair et al. 2004; Stasevich et al. 2010). However, the time scale of RICS experiments in the current study precluded detection of these binding processes, which occurred on the scale of seconds, as described by other workers (Carrero et al. 2004; Phair et al. 2004; Stasevich et al. 2010). On the other hand, an additional component of H1 mobility, characterized by a short correlation time (~3 ms), was present. This component may be attributed to movement (diffusion) of free H1 and is detectable only in the nuclear regions of low average H1 concentration. This notion is in line with the earlier results (obtained with FRAP) indicating that fraction of free H1 (Misteli et al. 2000) and the mobility of the H1 histone (Bancaud et al. 2009) are higher in euchromatin than heterochromatin. Correspondingly, the two RICS pattern types (clusters) were sufficient to describe the heterogeneity of H1 nuclear mobility. Attempts to isolate additional clusters did not reveal any additional components, but resulted in splitting of the existing clusters. One should note that the RICS window covered 2.65 × 2.65-µm regions, which are unlikely to be uniform in terms of H1 concentration and chromatin structure. Nonetheless, the two components of H1 mobility (diffusion and binding) were also detectable with a smaller window (down to 0.7 × 0.7 µm), albeit with a lower SNR (which precluded robust fitting).

Two types of H1 mobility were detectable with FCS as well. The shorter correlation time corresponded to diffusion of free histone, described by a standard 3D diffusion model. Moreover, the diffusion coefficient obtained in this study (*D* = 21.1 µm^2^/s) was consistent with the results obtained by others (Bhattacharya et al. 2006; Rao et al. 2007). However, the decay of ACF at longer times (>10 ms) was poorly described by the single-component model. We postulate that the decay represented a spectrum of processes with different characteristic times. This element of ACF might correspond to binding of H1, as suggested by other researchers (Bhattacharya et al. 2006; Rao et al. 2007). In accordance with RICS data, the former (diffusion) component was more pronounced in the nuclear regions of low H1 concentration, while the latter (binding) in the regions of high H1 concentration. The low SNR of the FCS data precluded exploring a possible dependence between the local H1 concentration and its mobility in detail. It should be noted that both fractions (mobile in FCS experiments) corresponded to only 20 % (low H1 concentration) or 10 % (high H1) of histone fluorescence (as estimated from the initial photobleaching). The remaining part was immobile on the scale of the FCS (and RICS) experiment (<10 s) and therefore may correspond to the stably bound H1 histone, as observed by others using FRAP (Carrero et al. 2004; Stasevich et al. 2010). It is interesting to note that heterochromatin protein 1 (HP1), which interacts with histone tails, also exhibits multiple patterns of mobility in nuclei (Schmiedeberg et al. 2004).

A decrease of translational H1 mobility in the regions of high H1 concentration is accompanied by a decrease of rotational mobility of the histone. This effect, albeit only a slight one, is not a product of measurement noise (as demonstrated with free eGFP as a control). However, the lowest rotational H1 mobility is detectable in the border areas between the regions of high and low H1 concentration. This notion could reflect the high rigidity of chromatin in these areas (Bhattacharya et al. 2009). Moreover, one might postulate that high concentration of H1 histone might not always correspond to condensed chromatin (heterochromatin), as was assumed earlier (Bancaud et al. 2009; Bhattacharya et al. 2006; Rao et al. 2007). This notion is corroborated by the imaging data, demonstrating the presence of condensed chromatin areas where the H1 concentration remains below the average value, proportional to a local DNA concentration. Conversely, some nuclear regions characterized by the opposite relationship were present. It should be noted that the local chromatin concentration (assessed with DAPI) many not fully reflect its compaction (manifested by various postulated higher order chromatin structures). Likewise, this parameter does not indicate the transcriptional state of chromatin. Nonetheless, taken together, these results indicate that some chromatin regions may pose obstacles to H1 mobility. The presence of these putative domains is detectable with pCF. These data indicate that some domains are inaccessible to H1 (on the time scale up 5 s), whereas a decrease of histone mobility (trapping) occurs in others. Moreover, the characteristic times corresponding to H1 mobility and trapping agree with FCS results. One should note that no barriers to free eGFP mobility in cell nuclei were detectable with pCF. Therefore, the putative H1 trapping might have been caused by its interaction with chromatin. However, no simple relationship between the presence of the areas inaccessible to the histone and its concentration could be derived from pCF measurements. This notion is in agreement with the anisotropy data, which indicate the presence of rigid chromatin domains characterized by a moderate H1 concentration. One may also hypothesize that these domains impede movement of H1 at larger distances, contributing to the low apparent diffusion coefficient detectable with FRAP.

## Conclusions and biological context

Translational mobility of molecules of H1.1 linker histone in cell nuclei was probed using a range of techniques, at small (1–100 µs, FCS, RICS, anisotropy), moderate (1–100 ms, FCS, RICS, pCF) and large (1–100 s, pCF and FRAP) time scales. The results indicate that the global mobility of H1 (at distances >1 µm) is determined by processes other than diffusion (ex. binding) that occur on a time scale of seconds. Hence, the apparent global diffusion coefficient of H1 (*D* ~ 0.02 µm^2^/s) is several orders of magnitude lower than the value predicted for an unimpeded movement. Moreover, a fraction (~25 %) of H1 is immobile at a time scale up to 50 s. The local H1 dynamics (at distances <0.7 µm) comprise diffusion and multiple binding processes occurring at longer time scales (10–2,500 ms). The diffusion predominates in the nuclear regions of low H1 concentration, whereas the binding dominates in the regions of high concentrations of the histone. Moreover, in some chromatin regions, a complete absence of H1 penetration or an impediment to its movement is observed. It is likely that these regions are characterized by low rotational mobility of H1 (high chromatin rigidity) and its concentration below the average value. These regions constitute the obstacles to movements of H1 at larger distances, thus contributing to a low value of the apparent global diffusion coefficient.

The measurements of mobility of H1 linker histone described above demonstrate that local determinations, based on FRAP or FCS alone, are unlikely to provide a comprehensive picture of the complexity of dynamics of this protein in various subnuclear regions. The general conclusion emerging from our FRAP and FRCS/RICS experiments is in agreement with the view that H1 histones bind to DNA transiently but show no specificity for any DNA sequence (Bustin et al. 2005; Catez and Hock 2010; Catez et al. 2006) and that the strength of binding to DNA may be moderated by local chromatin density (Bancaud et al. 2009). In other words, the residence time of H1 on DNA may, to some degree, be influenced by the molecular crowding phenomena. This notion is also in agreement with our previous observation of the influence of DNA intercalators on interactions between H1 linker histones and DNA (Wojcik and Dobrucki 2008; Wojcik et al. 2013). We demonstrated that a subpopulation of H1 is readily expelled from DNA of live cells by intercalating anthracyclines. However, a subpopulation of H1 seems to appear in heterochromatin, which remains in complex with DNA despite the presence of the drug, which is ready to compete with H1 for binding sites on DNA. The presence of multiple populations characterized by different kinetics of interaction with chromatin is not unique for H1 histones. It has been postulated that the dynamics of HP1 are affected by diverse modes of interaction with chromatin (Schmiedeberg et al. 2004). It is interesting to note that chromatin compaction is one of the factors influencing the HP1 dynamics.
